# The effect of hyperthyroidism on cognitive function, neuroinflammation, and necroptosis in APP/PS1 mice

**DOI:** 10.1186/s12967-023-04511-x

**Published:** 2023-09-22

**Authors:** Kai Lou, Shudong Liu, Fengxia Zhang, Wenxiu Sun, Xinhuan Su, Wenkai Bi, Qingqing Yin, Yaxin Qiu, Zhenyuan Zhang, Mengzhe Jing, Shizhan Ma

**Affiliations:** 1 Department of Endocrinology, Jinan Central Hospital Affiliated to Shandong First Medical University, Jinan, 250013 China; 2 Department of Endocrinology, Shandong Rongjun General Hospital, Jinan, 250013 China; 3 Department of Neurology, Affiliated Hospital of Shandong University of Traditional Chinese Medicine, Jinan, 250011 China; 4 Department of Nursing, Taishan Vocational College of Nursing, Taian, 271000 Shandong China; 5 Department of Geriatrics Endocrinology, Shandong Provincial Hospital Affiliated to Shandong First Medical University, Jinan, 250021 Shandong China; 6 Department of Endocrinology, Shandong Provincial Hospital Affiliated to Shandong First Medical University, Jinan, 250021 Shandong China; 7 Shandong Clinical Research Center of Diabetes and Metabolic Diseases, Jinan, 250021 Shandong China; 8 Shandong Key Laboratory of Endocrinology and Lipid Metabolism, Jinan, 250021 Shandong China; 9 Shandong Prevention and Control Engineering Laboratory of Endocrine and Metabolic Diseases, Jinan, 250021 Shandong China; 10 Department of Geriatric Neurology, Shandong Provincial Hospital Affiliated to Shandong First Medical University, Jinan, China

**Keywords:** Alzheimer’s disease, Graves’ disease, Single cell RNA-sequencing, Neuroinflammation, Necroptosis

## Abstract

**Background:**

Increasing evidence has linked the thyroid dysfunction to the pathogenesis of dementia. Evidence from clinical studies has demonstrated that hypothyroidism is related to an increased risk of dementia. But the association of hyperthyroidism with dementia is largely unknown.

**Methods:**

We used the adenovirus containing thyrotropin receptor (TSHR) amino acid residues 1-289 (Ad-TSHR289)-induced Graves’ disease (GD) phenotype in Alzheimer’s disease (AD) model mice (APP/PS1 mice) to evaluate the effect of hyperthyroidism on the cognitive function and β-amyloid (Aβ) accumulation.

**Results:**

GD mice exhibited a stable long-term hyperthyroidism and cognitive deficits. Single Cell RNA-sequencing analysis indicated that microglia function played a critical role in the pathophysiological processes in GD mice. Neuroinflammation and polarization of microglia (M1/M2 phenotype) and activated receptor-interacting serine/threonine protein kinase 3 (RIPK3)/mixed lineage kinase domain–like pseudo-kinase (MLKL)-mediated necroptosis contributed to the pathological process, including Aβ deposition and neuronal loss. RIPK3 inhibitor could inhibit GD-mediated Aβ accumulation and neuronal loss.

**Conclusions:**

Our findings reveal that GD hyperthyroidism aggravates cognitive deficits in AD mice and induces Aβ deposition and neuronal loss by inducing neuroinflammation and RIPK3/MLKL-mediated necroptosis.

**Supplementary Information:**

The online version contains supplementary material available at 10.1186/s12967-023-04511-x.

## Background

Thyroid hormones [THs: thyroxine (T4) and triiodothyronine (T3)] play a critical role in hippocampal neurogenesis, neuronal differentiation, and central nervous system function from early neurogenesis to brain maturation [1, 2]. Growing evidence demonstrated that thyroid dysfunction was associated with cognitive function and dementia, which reduced quality of life and has become a massive burden on health care systems worldwide [3–8]. Clinical and experimental studies have demonstrated a clear association between overt hypothyroidism and dementia, which overt hypothyroidism increased the risk of dementia by 12% for a 6-months period with high thyrotropin (TSH) levels [6, 9–11].

However, the association of hyperthyroidism with dementia seems controversial [12–15]. A large-scale registry-based data showed a higher incidence of dementia in individuals with hyperthyroidism including Graves’ disease and toxic nodular goiter, which GD is the most common cause of hyperthyroidism in aged patients [3, 12]. In contrast, another data analysis did not find the association between overt hyperthyroidism and dementia [15, 16].

Alzheimer’s disease (AD) is an age-related neurodegenerative disorder and the most common form of dementia, which includes AD, vascular dementia, and other dementia such as Parkinson’s disease, Lewy body dementia, frontotemporal dementia [8, 17]. AD-related dementia accounts for as high as 65% of total dementia people aged 60 years or older, as well as 27% vascular dementia and 8% other dementia according to a recent cross-sectional study in China. [8] The neuropathological features of AD include neuroinflammation, amyloid- β (A β) protein deposits, and neuron death, which cause clinical manifestation such as cognitive decline [18]. Accumulating evidence demonstrate that the pathological processes of AD are involved in activated microglia and necroptosis, neurotoxicity, mitochondria defects [19, 20]. Microglia can exhibit a proinflammatory (M1) phenotype or an anti-inflammatory (M2) phenotype, which the phenotype change of active microglia from M2 polarization to M1 could influence the Aβ phagocytosis, inflammatory state, and brain homeostasis [21]. Prior studies revealed that T3 affects macrophage differentiation and function inducing M1 polarization and reversing M2 polarization [22, 23]. However, the effect of hyperthyroidism on microglia polarization remains to be elucidated.

Necroptosis is a process responsible for regulating cell death in response to microenvironmental changes, and an important contributor to pathogenesis of AD [20, 24, 25]. Once necroptosis is activated, three key mediators such as receptor-interacting serine/threonine protein kinase (RIPK) 1, RIPK3, and mixed lineage kinase domain–like pseudokinase (MLKL), sequentially undergo phosphorylation modification and recruited for a necrosome leading to neuron death and neuroinflammation [20, 26, 27]. Necroptosis is activated in brains of AD human and AD mice, and suppression of necroptosis exerts a neuroprotective effect on cognitive impairment in AD mouse model: 5xFAD and APP/PS1 mice [20, 28–30]. A previous study demonstrated that T3 could induce necroptosis activation in the retina suggesting a role for thyroid hormone in necroptosis [31].

To best investigate the role of hyperthyroidism in affecting cognitive function and microglia polarization, as well as necroptosis, a long-term hyperthyroidism of GD mouse model were established through injecting recombinant adenovirus overexpressing thyrotropin receptor A subunit 289 (Ad-TSHR289) in AD model mice (APP/PS1) [32–34]. So far, there is no effective treatment strategy to eliminate the pathological changes in the brain and restore cognitive function in AD patients. Elucidation the effect of hyperthyroidism on cognitive function and its underlying mechanism might strengthen the understanding the thyroid hormone function and provide help for early-stage prevention of hyperthyroidism induced-dementia.

## Methods

### Animal treatment

The adenovirus expressing amino acid residues 1-289 of TSHR (Ad-TSHR289) and adenovirus-control (Ad-Control) were purchased from Hanbio Biotechnology Co., Ltd. (Shanghai, China). 10-week-old female specific pathogen-free (SPF) APP/PS1 mice with a C57BL/6J background were purchased from JunKe Biological Co., Ltd. (Nanjing, China). All the mice were kept in an SPF room with controlled lighting (12 h on, 12 h off), at a temperature of 22–25 °C. All experiments were conducted in accordance with the principles and procedures outlined in the Guideline for the Care and Use of Laboratory Animals and were approved by the Ethics Committee of Shandong Provincial Hospital affiliated with Shandong First Medical University (NO. 2019 − 131).

The immunization protocols for GD animal models were performed as previously described including the initial stage (3 injections) and maintenance stage (6 injections) [33]. After 2 weeks of adaptive feeding, the mice were randomly divided into two groups: the APP/PS1 group (n = 20) and APP/PS1 + GD group (n = 12). The APP/PS1 + GD mice were immunized and injected into the left and right femoral muscles with 50 µl phosphate-buffered saline (PBS) containing Ad-TSHR289 particles up to 9 times at the 0, 3rd, 6th ,10th ,14th ,18th ,22nd ,26th, and the 30th week, respectively [33]. The APP/PS1 mice were injected with the same dose of an adenovirus vehicle.

As for treatment, the mice were randomized into three groups: the APP/PS1 group (n = 32), the APP/PS1 + GD group (n = 24), and APP/PS1 + GD + GSK872 group (n = 24).The mice in the APP/PS1 group were injected with 50 µl PBS containing Ad-vehicle, and the mice in the APP/PS1 + GD group were injected with Ad-TSHR289 in combination with saline (vehicle). In APP/PS1 + GD + GSK872 group, the potent selective RIPK3 inhibitor GSK872 was diluted to1 mg/ml in vehicle (saline). At the 28th week, mice were administrated by intraperitoneal injection with GSK872 (1 mg/kg) every day for 6 weeks. Blood was harvested from the tail vein at the 6th, 11st, 23rd, and the 34th week for analysis. Necrostatin-1s (Nec-1s; a specific inhibitor of RIPK1) was administered according to GSK872 and a previously reported method [35].

### Analysis of single-cell transcriptomics data

Single-cell FASTQ sequencing reads from each sample were processed and converted to digital gene expression matrices after being mapped to the reference genome using the CellRanger Single Cell Software Suite (v3.1.0) on the 10x genomics website [36]. These datasets were analysed by the R package Seurat (v 3.1.0) [37]. Cells expressing fewer than 200 genes were removed from the dataset. The number of genes, UMI counts and percentage of mitochondrial genes were examined to identify outliers. As an unusually high number of genes can result from a ‘doublet’ event, in which two different cell types are captured together with the same barcoded bead, cells with > 90% of the maximum genes were discarded. Cells containing > 7.5% mitochondrial genes were presumed to be of poor quality and were also discarded. The gene expression values then underwent library-size normalization, in which raw gene counts from each cell were normalized to the total number of read counts present in that cell. The resulting expression values were then multiplied and log-transformed. Subsequent analyses were conducted using only the most highly variable genes in the dataset.

Principal component analysis was used for dimensionality reduction, followed by clustering in principal component analysis space using a graph-based clustering approach. Uniform manifold approximation and projection (UMAP) was then used for two-dimensional visualization of the resulting clusters. For each cluster, the marker genes were identified using the FindConservedMarkers function in the Seurat package (logfc.threshold > 0.25 and minPct > 0.25). Then, clusters were assigned to a known cell type according to the Cell Marker database. [38] Differentially expressed genes (DEGs) across different samples were identified using the FindConservedMarkers function in Seurat using the parameters logfc.threshold > 0.25, minPct > 0.25, and Padj ≤ 0.05’. STRING analysis [39] was performed DIAMOND(v0.8.31) [40] to assess the interactions between proteins encoded by the DEGs. Gene Ontology (GO) analysis and KyotoEncyclopedia of Genes and Genomes (KEGG) pathway analysis were performed using phyper, a function of R. Only GO terms or KEGG pathways with an FDR < = 0.05 were considered significantly enriched.

### Statistical analysis

Values are presented as the means ± SEMs. The significance of differences between two groups was analysed by unpaired Student’s *t*-test, one-way ANOVA analysis of variance for multiple comparisons using GraphPad Prism 6.0 (GraphPad Software Inc, San Diego, CA, USA). P < 0.05 was considered significant.

## Results

### GD aggravates cognitive deficits in APP/PS1 mice

**Fig. 1:**
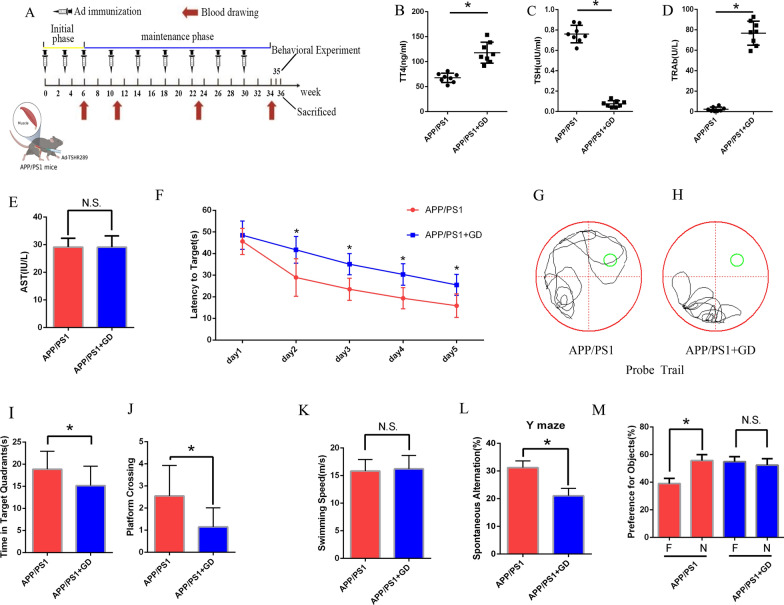
GD aggravates cognitive deficits in APP/PS1 mice. **A** Schematic diagram of the study. APP/PS1 mice were injected with Ad-TSHR289 or Ad-vehicle (n = 32). **B**–**E** The serum levels of TT4 (**B**), TSH (**C**), TRAb (**D**), and AST (**E**) were measured at 34th week after the 1st injection. **F** Latency to target in the MWM test (n = 6/group). **G**, **H** Representative tracings of APP/PS1 mice (**G**) and GD model mice (**H**) in the probe trial. **I**, **J** The time spent in the target quadrant (**I**) and the number of crossings the platform (**J**) in the probe trial. **K** Swimming speed in the MWM test. **L** The Y maze spontaneous alternations (n = 6/group). **M** Novel object recognition test (n = 6/group). *F* familiar object; *N* novel object

To determine whether GD is associated with cognitive function, Ad-TSHR289 was initially injected into the 3-month-old APP/PS1 mice to establish a GD model as previously described (Fig. 1A) [32, 33]. At 6th, 11st, 23rd, 34th week, blood was collected for measurement of total thyroxine (TT4), TSH, TSH receptor antibody (TRAb), and aspartate aminotransferase (AST) levels. After injection of Ad-TSHR289 into APP/PS1 mice, the mice presented significantly elevated levels of serum TT4 and TRAb with a decreased TSH levels at 34th week(others data not shown), accompanying with hypercellularity and hypertrophy of thyroid follicles suggesting that the APP/PS1 mice exhibited a GD phenotype (Fig. 1B and D, Additional file 1: Fig. S1A). There was no significant difference in AST levels in GD mice compared to the APP/PS1 mice (Fig. 1E). Magnetic resonance imaging (MRI) was performed to evaluate anatomic changes in the mouse brain. No obvious difference was found in MR signals intensity between the brains of GD model mice and those of APP/PS1 mice (Additional file 1: Fig. S1B).

To further determine whether GD influenced cognitive function, we assessed the cognitive function of mice by the behavioral experiment such as morris water maze (MWM) test. MWM training trials were performed on five consecutive days at the 35th week. The latency of mice in the GD group was significantly longer compared to APP/PS1 mice after day 2 (Fig. 1F). In the probe trial, the GD mice exhibited a longer distance travelled to reach the platform compared to APP/PS1 mice (Fig. 1G, H). GD mice in the group spent a significantly shorter time in the target quadrant and had a decreased the number of platform crossings (Fig. 1I, J). However, there was no significant difference in swimming speed between the GD mice and APP/PS1 mice (Fig. 1K).

GD mice exhibited decreased spontaneous alternation behaviour in the Y-maze test, which was used to evaluate short-term memory (Fig. 1L). In the novel object recognition test, GD mice showed an impaired ability to recognize the novel object (Fig. 1M). All these results indicate that GD aggravates cognitive deficits in APP/PS1 mice.

### GD increases Aβ accumulation and neuronal loss in APP/PS1 mice

**Fig. 2:**
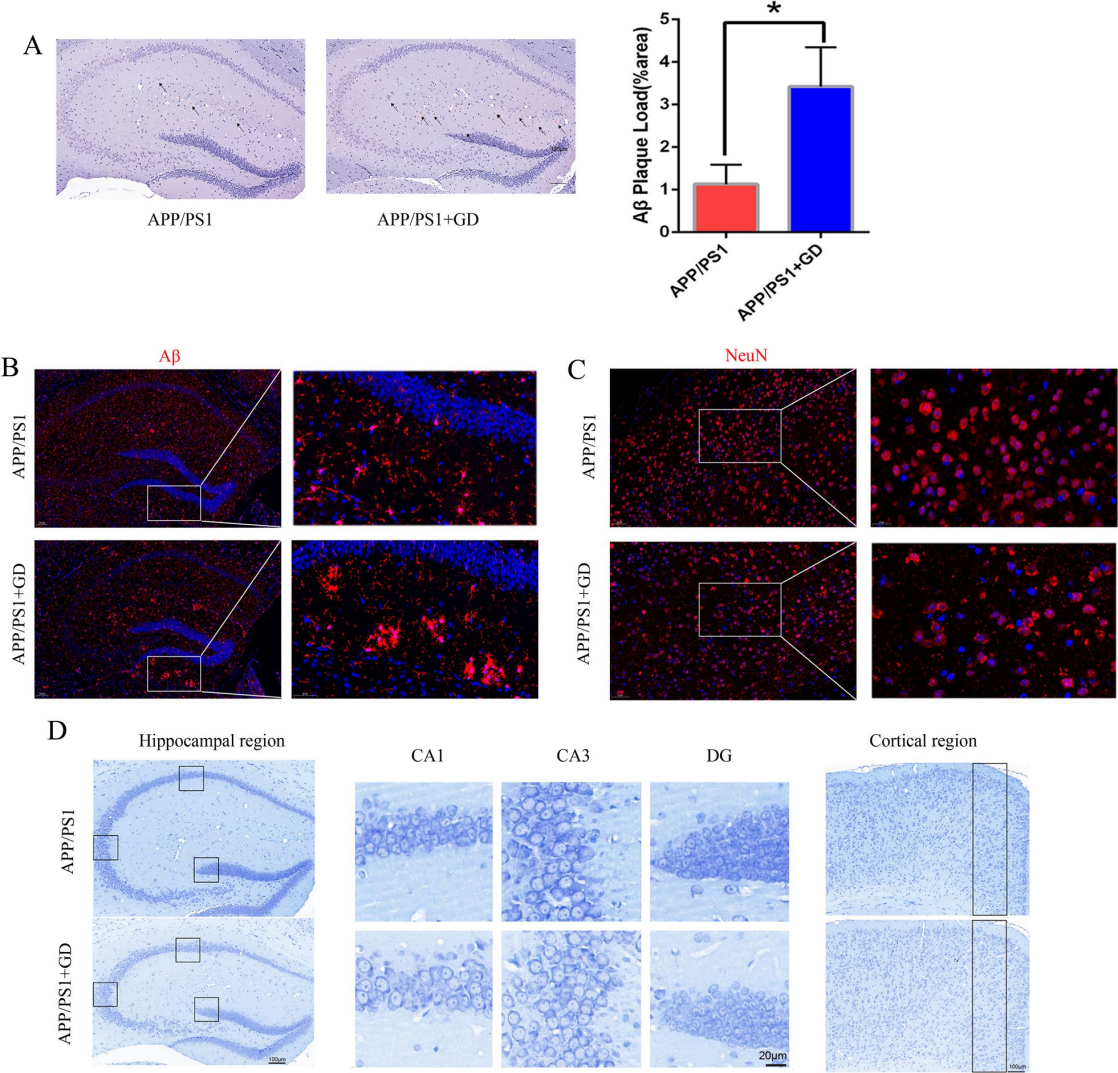
GD increases Aβ accumulation and neuronal loss in APP/PS1 mice. **A** Congo red staining of Aβ plaque (arrow) and quantification in the hippocampus. Scale bar = 200 μm. **B** Immunostaining of Aβ protein at higher magnification at hippocampus of the mice (n = 4). Scale bar, 100 μm and 50 μm. **C** Representative images of NeuN immunostaining at higher magnification in the cortex of the mice (scale bar, 50 μm and 20 μm). **D** Representative images of Nissl staining in the hippocampus (scale bar, 100 μm and 20 μm) and cortex (scale bar, 100 μm) of the indicated mice. Unpaired Student’s *t*-test, *p < 0.05 compared to APP/PS1 mice. The values are represented as mean ± SEM (n = 6/group). *N.S*. no significance

To analyse the neuroanatomical changes underlying the cognitive decline in GD model mice, Congo red staining method was employed to determine Aβ plaques in the hippocampus. Histological analysis showed that Aβ plaque load significantly increased in the hippocampus of the GD mice relative to APP/PS1 mice (Fig. 2A). Similarly, immunostaining for Aβ of the hippocampus also showed that Aβ protein load was significantly increased in GD model mice (Fig. 2B, Additional file 1: Fig. S2A).

Furthermore, we determined whether GD induced brain neuronal loss. Neuronal nuclear antigen (NeuN) is widely used as a marker of neurons in the central nervous system. [41] Immunostaining for NeuN consistently showed an obvious decrease in the number of NeuN-positive cell in the cortical region but not in the hippocampus in the GD mice compared to the APP/PS1 mice (Fig. 2C, Additional file 1: Fig. S2B, and C). Nissl staining in GD model mice represented greater neuronal loss at the cortical region compared to APP/PS1 mice. However, there were no differences in neuronal loss in the CA1, CA3 regions, and ordentate gyrus (DG) of the hippocampus (Fig. 2D, Additional file 1: Fig. S2D).These findings suggest that GD increases Aβ accumulation and neuronal loss in APP/PS1 mice.

### Single-cell RNA sequencing analysis of hippocampal tissues

**Fig. 3:**
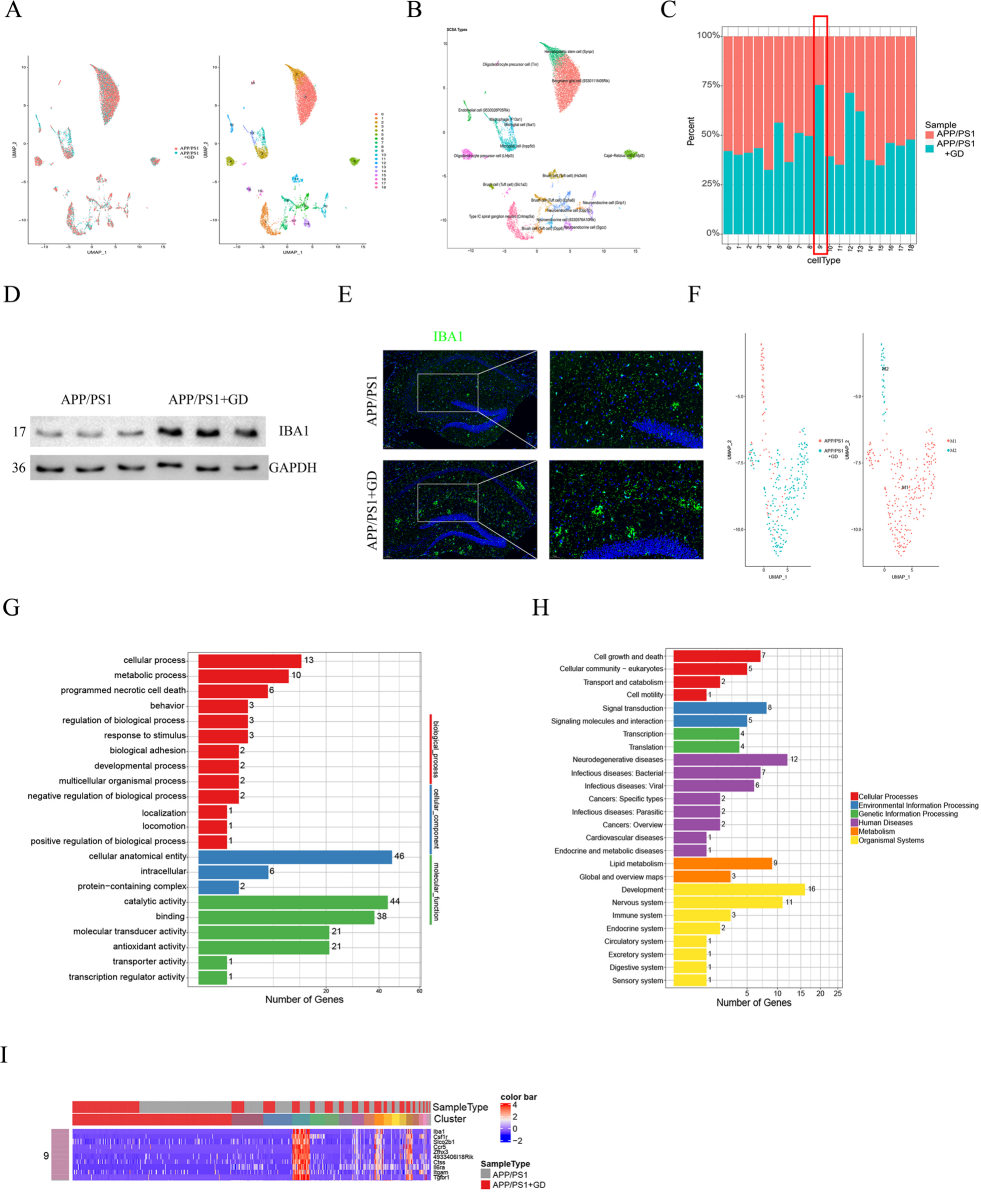
Single-cell RNA-sequencing analysis of hippocampal tissues. **A** UMAP plot of 11,366 mouse hippocampal cells isolated from GD mice and APP/PS1 mice. **B** Annotations of the cell types from single-cell RNA sequencing data by SASC. **C** The bar chart shows the percentage of each cell type in the hippocampus of GD mice and APP/PS1 mice. **D** Western blotting analysis of the IBA1 protein and quantification in the hippocampus of mice. **E** Immunostaining of IBA1 at higher magnification and quantification of the IBA1-positive cell density in the hippocampus of mice. Scale bar, 100 μm and 50 μm. Unpaired Student’s *t*-test, *p < 0.05 compared to control mice. The values are represented as mean ± SEM (n = 5/group). **F** UMAP plot of cluster 9 (microglia) revealing a change in M1/M2 polarization. **G** Bar chart shows the results of GO enrichment analysis of the microglial DEGs identified using single-cell RNA sequencing. **H** Bar chart showing that microglial DEGs is enriched in the KEGG pathways. The number of DEGs involved in the pathways is shown. **I** Truncated heatmap showing the top 10 DEGs in microglial cells (cluster 9)

Single-cell RNA sequencing is a valuable approach for investigating the physiological and pathophysiological mechanisms of neurodegenerative diseases [42]. We performed single-cell RNA sequencing of hippocampal tissues from GD mice and APP/PS1 mice to identify cells and genes that may contribute to GD. A total of 11,366 mouse hippocampal cells including 6376 cells from control mice and 4990 cells from GD model mice were analysed, and 19 cell clusters (types) were delineated, which were comparable between groups (Fig. 3A). Cell type annotation using SCSA showed that the predominant cells were neuroendocrine cells and microglial cells (cluster 9), which ionized calcium binding adapter molecule 1 (IBA1) uses as a microglia-specific marker(Fig. 3B) [43]. Moreover, the data indicated that microglia (cluster 9) accounted for approximately 75% of all cells and were the majority of cells in the hippocampus of GD model mice (Fig. 3C).To confirm these findings, we measured IBA1 protein levels in the hippocampus of GD mice and APP/PS1 mice. There was a significant increase in the protein expression of IBA1 in GD mice compared to APP/PS1 mice (Fig. 3D and E, Additional file 1: Fig. S3A, B). These data suggests that microglia is involved in the pathophysiological process of GD-related cognitive decline.

### GO enrichment analysis and KEGG pathway analysis of DEGs in the hippocampus

Microglia can exhibit two different phenotypes (the M1 phenotype and M2 phenotype), which differ in function in response to different stimulus. Since single cell RNA-sequencing indicated that microglia were involved in pathophysiological processes in GD model mice. Thus, we next determined the phenotype of microglia in cluster 9 based on the expression of M1 and M2markergenes [44]. The data showed that the predominate microglia phenotype was the M1 phenotype and that M2 polarization was inhibited in the hippocampus of GD mice compared to those of control mice (Fig. 3F).

To better elucidate the effect of the change in microglial phenotype, DEGs in microglia were subjected to GO enrichment analysis and KEGG pathway analysis. GO terms include biological processes, cellular components, and molecular functions. The results indicated that the DEGs were most highly enriched in biological processes such as cellular processes, metabolic processes, and necroptosis. Regarding cellular components, the 46 DEGs were enriched in cellular anatomical entities. Regarding molecular function, most of the DEGs were enriched in catalytic activity, binding, molecular transducer activity, and antioxidant activity (Fig. 3G).

KEGG pathway analysis showed that the DEGs were enriched in six major pathways, including cellular processes such as cell growth and death (15 genes), environmental information processing (13 genes), genetic information processing (8 genes), human diseases (33 genes), metabolism (12 genes), and organismal systems (36 genes). Notably, the enriched pathways of 12 DEGs were associated with human neurogenerative diseases (Fig. 3H). Subsequent analysis revealed that the top DEGs in microglial cells, such as chemokine (C-C motif) receptor 5 (Ccr5), integrin alpha-M (Itgam), colony-stimulating factor 1 receptor (CSF1R), and interleukin-6 receptor subunit alpha (Il6ra), which are implicated in AD, were involved in inflammation or microglial development (Fig. 3I and Additional file 1: Fig S3C) [45–48]. These results confirmed that microglial function plays a critical role in the pathophysiological process in GD model mice.

### GD affects the microglial polarization in the hippocampus of APP/PS1 mice

**Fig. 4:**
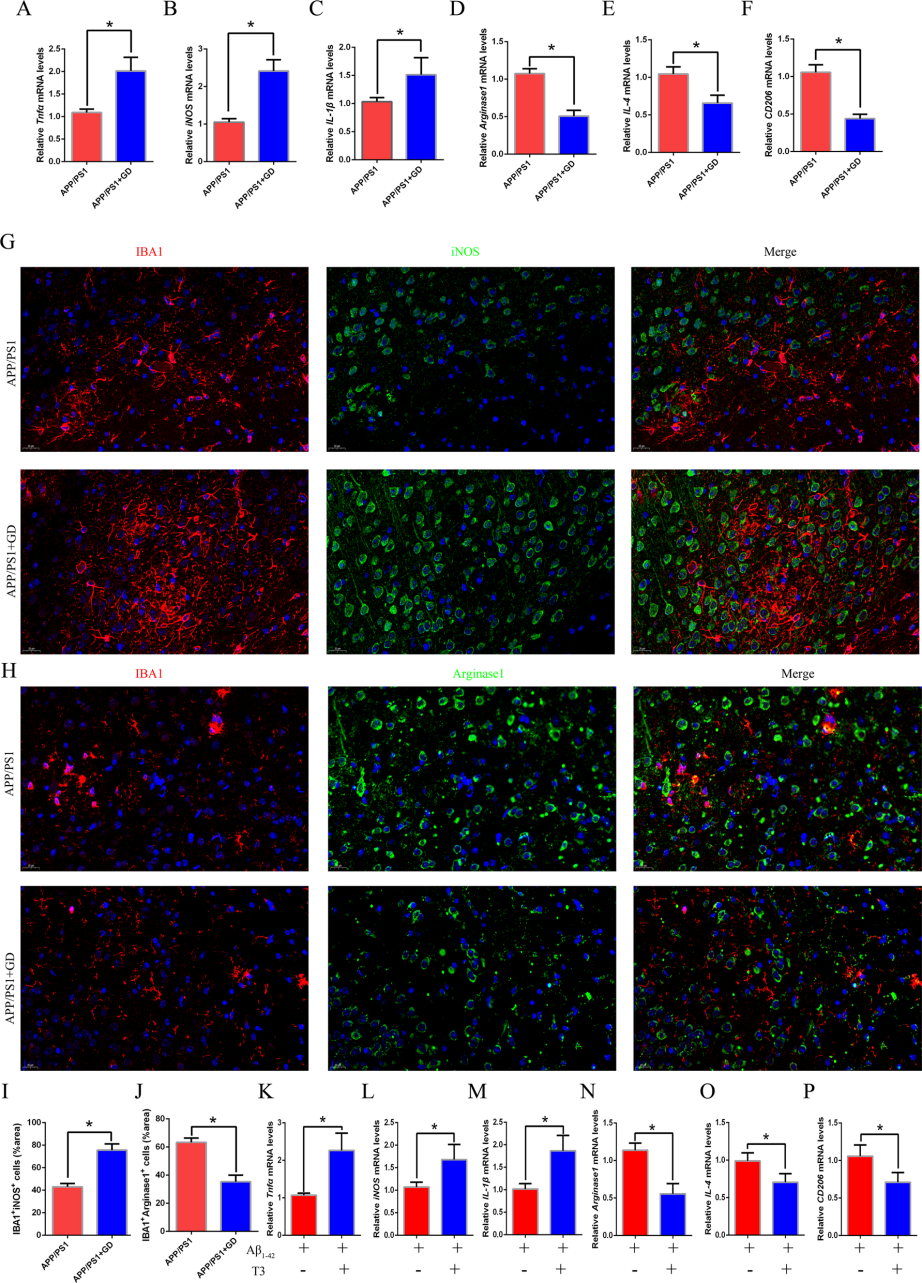
GD affects the microglial polarization in the hippocampus of APP/PS1 mice. **A**–**C** The mRNA expression of M1 phenotype markers TNFα (**A**), iNOS (**B**), and IL-1β (**C**) in the hippocampus.(**D**–**F**) The mRNA levels of the M2 phenotype markers Arginase 1 (**D**), IL-4 (**E**), and CD206 (**F**) in the hippocampus. **G** Double immunostaining of iNOS and IBA1 in the hippocampus in mice. Scale bar, 20 μm. **H** Double immunostaining of Arginase 1 and IBA1 in the hippocampus. Scale bar, 20 μm. **I** Quantification of the number of IBA1/iNOS double-positive cells in (**G**). **J** Quantification of the number of IBA1/Arginase1 double-positive cells in (**H**). Unpaired Student’s *t*-test, *p < 0.05 compared to control mice. The values are represented as mean ± SEM (n = 5/group). **K** Primary microglia from APP/PS1 mice were treated with oligomeric Aβ_1–42_ and then treated with or without T3. The mRNA levels of TNFα (K), iNOS (**L**), IL-1β (**M**), Arginase 1 (**N**), IL-4 (**O**), and CD206 (**P**) were measured. Unpaired Student’s *t*-test, *p < 0.05 compared to microglia without T3 treatment. The values are presented as the mean ± SEM from three independent experiments

M1/M2 polarization of microglia exerts a profound effect and inflammatory response in AD process [49]. Since we observed a change in microglial phenotype in GD mice, then we examined the related cytokines to confirm that GD influence microglial polarization to the M1 phenotype. Compared to control APP/PS1 mice, the specific markers of M1 microglia including tumour necrosis factor α (TNFα), iNOS, and interleukin-1 β (IL-1 β) were significantly increased in the hippocampal region in GD model mice, suggesting that GD can promote microglial polarization to the M1 phenotype (Fig. 4A, C). At the same time, M2 microglia markers including the mRNA levels of Arginase 1, IL-4, and CD206 were also measured. The results showed marked decreases in Arginase 1, IL-4, and CD206 levels in GD model mice (Fig. 4D, F).

In order to confirm the alteration of microglial phenotype, we examined the colocalization of iNOS/ Arginase 1 and IBA1 by double immunostaining. Immunostaining revealed that the iNOS was highly expressed in the microglial marker IBA1positive regions in GD mice (Fig. 4G and I). However, Arginase 1 expression and the number of IBA1/Arginase 1 double-positive cells were significantly decreased in the hippocampus of GD mice (Fig. 4H, J).

To determine whether exogenous T3 affects microglial inflammation and polarization in vitro, primary microglial cells from the brains of APP/PS1 mice were exposed toAβ_1–42_with or without T3, as oligomeric Aβ_1–42_ can induce microglial neuroinflammation and contribute to AD pathology [50, 51]. Similarly, T3 treatment increased the proinflammatory response (TNFα, iNOS, and IL-1β levels) and decreased the anti-inflammatory response (Arginase 1, IL-4, and CD206 levels) (Fig. 4K and P). Collectively, these results in vivo and in vitro demonstrate that GD affects the neuroinflammation and polarization of microglia from the M2 phenotype to the M1 phenotype in the hippocampus in APP/PS1 mice.

### GD induces the activation of necroptosis in the hippocampus of APP/PS1 mice

KEGG analysis showed that 6 DEGs were associated with necroptotic process (Fig. 3G). Extensive research has shown thatRIPK1/RIPK3, as serine/threonine-protein kinases, can autophosphorylate themselves and form a RIPK1/RIPK3 complex (necrosome) to recruit and induce the phosphorylation of MLKL at ser358 (p-MLKL) and form RIPK3-MLKL oligomers, which subsequently activate cell necroptosis to induce membrane rupture and cell death and thus play an important role in AD pathogenesis [20, 35, 52–55]. We then determine whether the RIPK/MLKL-induced necroptosis is activated in GD model mice.

**Fig. 5:**
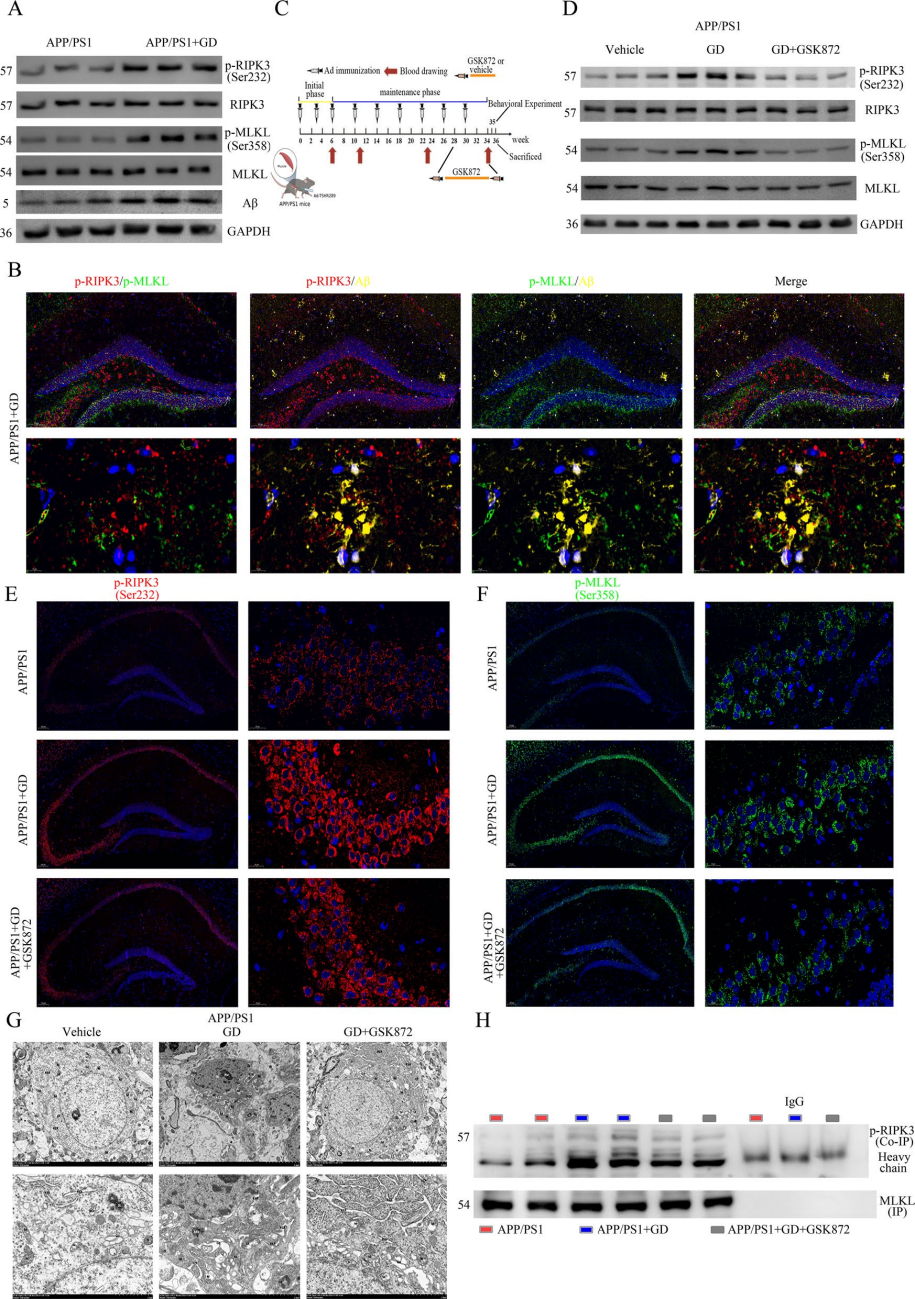
GD induces the necroptosis activation in the hippocampus of APP/PS1 mice. **A** Western blotting analysis of RIPK3/MLKL and Aβ in the hippocampus of mice. **B** (Top) Representative images of coimmunostaining of p-RIPK3 (red), p-MLKL (green), and Aβ (yellow) in the hippocampus of GD model mice. Scale bar, 100 μm. (Bottom) Higher magnification images. Scale bar, 20 μm. **C** Schematic drawing of the experimental schedule: APP/PS1 mice were treated with Ad-TSHR289 or Ad-Control in the presence of either vehicle or GSK872. **D** Western blotting analysis of p-RIPK3 and p-MLKL levels in the hippocampus of the indicated mice. **E** Immunostaining of p-RIPK3 at higher magnification in the hippocampus of the indicated mice. Scale bar, 200 μm. **F** Immunostaining of p-MLKL at higher magnification in the hippocampus of mice. Scale bar, 200 μm. **G** TEM of the hippocampus of the indicated mice. Scale bar, 5 μm or 2 μm. **H** Immunoprecipitation was performed to assess the interaction of p-RIPK3 with MLKL

To explore whether RIPK1-dependent signalling mediates the activation of necroptosis in the hippocampus of GD model mice, we delivered either vehicle or Nec-1s (a specific inhibitor of RIPK1) to GD model mice and APP/PS1 mice. Nec-1s treatment did not affect p-MLKL levels in GD model mice (Fig S4A). Though there were no differences in the total RIPK3 and MLKL proteins levels between GD model mice and control mice, the levels of activated RIPK3 (p-RIPK3; ser232), p-MLKL, and Aβ deposition were significantly increased in the hippocampus of GD model mice (Fig. 5A, Additional file 1: Fig. S4C). On the other hand, triple immunofluorescence showed that the p-RIPK3 but not p-RIPK1 was colocalized with IBA1 in the hippocampus (Additional file 1: Fig S4B). These data indicate that RIPK3, but not RIPK1, is involved in activating microglia necroptosis in hippocampus of GD model mice.

To further evaluate whether necroptosis affects the Aβ deposition in GD mice, we performed triple immunofluorescence staining of hippocampal tissues. Immunofluorescence revealed that p-RIPK3 and p-MLKL were colocalized with Aβ plaques (Fig. 5B), confirming that GD induces necroptosis activation and Aβ deposition in AD mice.

To further validate the necessity of p-RIPK3 on necroptosis activation, GD model mice were intraperitoneally administered GSK872 (a RIPK3 kinase inhibitor) every day for 4 weeks (Fig. 5C). GSK872 treatment did not affect TT4, TSH, TRAb, or AST levels in GD model mice, suggesting that it has no effect on GD pathology (Additional file 1: Fig S4D-4G).

Western blotting analysis and immunofluorescence were performed to quantify necroptosis-related protein levels. There was a significantly increased p-RIPK3 and p-MLKL expression in the hippocampus of GD model mice, and GSK872 treatment attenuated this change and profoundly reduced the protein expression of p-RIPK3 and p-MLKL(Fig. 5D, F, Additional file 1: Fig. S4H–4J). These results indicate that RIPK3 plays a critical role in necroptosis activation in GD mice.

The morphological changes in the mouse hippocampus were observed by transmission electron microscopy (TEM). The result showed that the three groups of neuronal cells underwent different degrees of damage. Neuronal cell damage, including pyknosis, increased nuclear chromatin and cytoplasm electron density, cell membrane breakdown, cell body shrinkage, mitochondria swelling, cristae disappearance, and perinuclear space widening, was aggravated in GD model mice compared to control mice. GSK872 treatment significantly alleviated this damage (Fig. 5G).

Thereafter, we performed immunoprecipitation to examine physiological relevance of the RIPK3 and MLKL. The amount of p-RIPK3 bound to MLKL was significantly higher in GD model mice than in control mice, and the change was rescued in GSK872-treated mice (Fig. 5H). In summary, these results demonstrate that GD induces the activation of necroptosis in the hippocampus in APP/PS1 mice mainly by inducing RIPK3/MLKL activation and interaction.

### GD-induced RIPK3 activation affects Aβ levels in the hippocampus of APP/PS1 mice

**Fig. 6:**
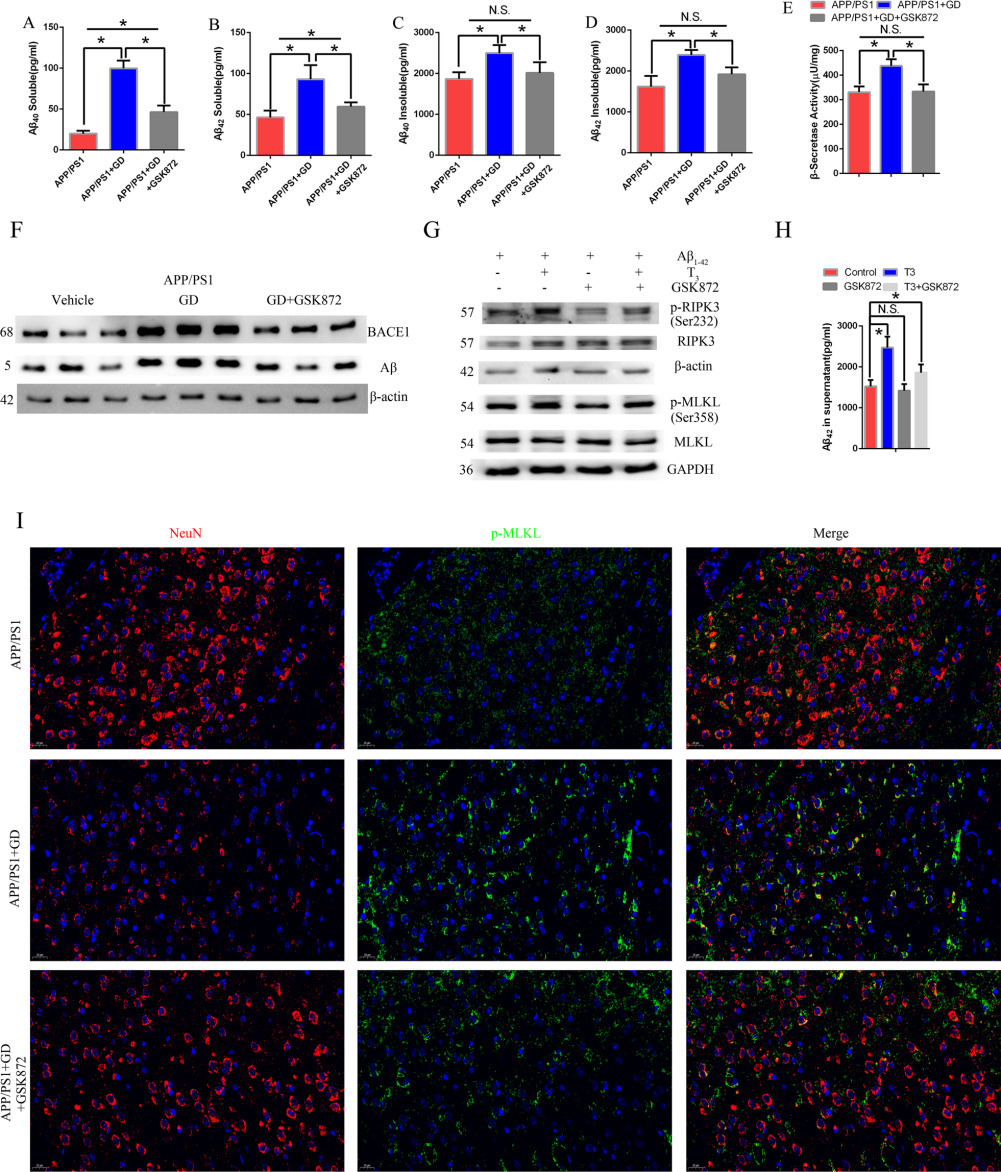
GD-induced RIPK3 activation affects Aβ levels and increases neuronal loss in the APP/PS1 mice. **A** The levels of soluble **A** and **B** and insoluble **C** and **D** Aβ_40_ and Aβ_42_ in mouse hippocampal samples measured by ELISA. **E** β-Secretase activity in the hippocampus of mice. **F** Western blotting analysis of BACE1 and Aβ expression in the hippocampus in the indicated mice. One-way ANOVA followed by Tukey test. *p < 0.05. The values are presented as the mean ± SEM (n = 6/group). **G** Primary microglia from APP/PS1 mice were treated with oligomeric Aβ_1–42_ and then treated with T3 or GSK872. Western blotting analysis was used to examine RIPK3, MLKL, p-RIPK3, and p-MLKL protein levels. **H** The levels of Aβ_42_ in the supernatant were evaluated by ELISA. One-way ANOVA followed by Dunnett’s test. *p < 0.05 compared to control cell. The values are presented as the mean ± SEM. N.S.: no significance. **I** APP/PS1 mice were treated with Ad-TSHR289 or Ad-Control in the presence of either vehicle or GSK872. Double immunostaining of p-MLKL and NeuN in the cortex

Since GD-induced RIPK3 activation is involved in AD pathogenesis, we further investigated the underlying mechanism and whether GD affects Aβ metabolism. The levels of soluble and insoluble Aβ_40_ and Aβ_42_ in the hippocampus were measured by ELISA. In line with the data presented above, there was a significant increase in the levels of both soluble and insoluble Aβ_40_ and Aβ_42_ in GD model mice, while GSK872 treatment reversed GD-induced Aβ accumulation (Fig. 6A and D). BACE1 is the rate-limiting enzyme for Aβ generation and has become a promising therapeutic target for AD treatment [56, 57]. We found a significant increase in Aβ and BACE1 protein levels and β-secretase activity in the hippocampus in GD model mice compared with control mice, which was attenuated by the RIPK3 kinase inhibitor (Fig. 6E, F, Additional file 1: Fig. S4K).Together, these data suggest that RIPK3-induced necroptosis contributes to the increase in Aβ generation.

We next evaluated the effect of RIPK3/MLKL activation on microglial Aβ phagocytosis. Primary microglial cells from APP/PS1 mice were exposed to oligomeric Aβ_1–42_ and then treated with or without T3 in the presence of either vehicle or GSK872. The p-RIPK3 and p-MLKL levels were significantly increased in T3-treated cells compared to only Aβ_1–42_-treated cells (control), while GSK872 treatment alleviated this alteration (Fig. 6G, Additional file 1: Fig. S4L) [58]. We found that T3-treated microglial cells eliminated Aβ more slowly than control microglial cells, while GSK872 antagonized the effect of T3 suggesting a decrease in the phagocytic activity of T3-treated microglia can be rescued by RIPK3 inhibition (Fig. 6H). Taken together, these results indicate that GD-induced necroptosis can affect Aβ metabolism during AD pathogenesis.

### GD activates necroptosis and increases neuronal loss in the cortex of APP/PS1 mice

The above results revealed that GD increases neuronal loss in the cortical region but not the hippocampal region in mice (Fig. 2D). We then determined whether RIPK3/MLKL-mediated necroptosis is associated with neuronal loss in cortex of GD model mice. Immunofluorescence staining revealed an obvious decrease in the NeuN-positive area accompanied by an increase in p-MLKL levels in GD model mice, while GSK872 treatment decreased p-MLKL levels and increased the NeuN- neurons area, and improved behavioral performance in the MWZ compared to GD model mice (Fig. 6I, Additional file 1: Fig. S4M–S4N, S5). These data indicate that GD-induced neuronal loss is involved in RIPK3/MLKL-activated necroptosis, and inhibition of RIPK3 relieves neuronal loss in the cortex of APP/PS1 mice with GD.

## Discussion

GD accounts for the vast majority of the hyperthyroidism, which affects energy metabolism, cardiovascular system, sympathetic nervous system, liver, and eyes leading to variable and nonspecific clinical feature [59, 60]. But, some hyperthyroidism patients often neglect systematic treatment owing to mild symptoms, easy recurrence, and adverse effect of thionamides, which lead to various complications under the state of long-term hyperthyroidism [59]. Evidence indicates that cognitive deficit is associated with GD. [14, 61]. The pathogenic mechanisms of cognitive deficit and hyperthyroidism are currently unknown. Thus, it is important to clarify the effect and mechanism of long-term hyperthyroidism on non-classic target glands, such as brain, which is helpful for us to improve and manage hyperthyroidism at an earlier stage in hyperthyroidism patients.

In this study, we focused on the effect of GD hyperthyroidism on cognitive function. To fulfil the purpose, the 3-month-old female APP/PS1 transgenic mice were immunized and injected with Ad-TSHR289 to induce the GD phenotype, which was a well-established animal model for GD [32, 33, 62]. Previous study has demonstrated that GD model mice exhibited a significant increase in TRAb and T3 levels from 3 weeks to 17 weeks after single immunization with Ad-TSHR289, and displayed stable hyperthyroidism 9 weeks at least to avoid the transient effect of THs on cognitive function [32]. It is worth noting that there is a gender difference in the establishment of the animal model of GD, with females being more predisposed to autoimmune diseases. There was a higher incidence of hyperthyroidism in female mice established an experimental animal model of GD [63–66]. On other hand, since the incidence of GD and AD is much higher in females than in males, we chose female mice as the research object of this study [8, 67, 68]. GD model mice in this study exhibited higher TT4, TRAb levels, lower TSH levels for 28 weeks, and represented behavioural impairment, and aggravation of cognitive decline such as spatial learning and memory dysfunction. Frank and colleagues found that nondemented elderly subjects with higher FT4 levels had more hippocampal and amygdalar atrophy on MRI, which can be an early indicator of incipient AD [69]. However, we did not find changes in the brains of GD model mice on MRI, this inconsistency is probably because the participants of the study by Frank and colleagues were ageing and exhibited high FT4 levels for 5.5 years.

Aβ accumulation and neuronal loss in the brain are cardinal features of AD pathophysiology [18, 24, 25]. We observed significant aggravation of Aβ accumulation in the hippocampus of GD model mice, confirming a direct linkage between GD and AD. Interestingly, our results showed a decrease in the number of neurons in the cortex of GD model mice, but there was no significant difference in the number of neurons in the CA1 and CA3 regions or DG of the hippocampus. Why GD induces a higher degree of neuronal death in the cortex but not in the hippocampus needs to be further studied.

We performed single-cell RNA sequencing of hippocampal samples from GD model mice and control AD mice to determine which mediators or cells contribute to the Aβ accumulation and neuronal loss. Single-cell RNA sequencing analysis showed that the cluster 9 was identified as microglia based on a higher expression of its specific marker IBA1and CSF1R in GD model mice suggesting that microglia was likely involved in the pathophysiological process of GD-related cognitive decline. Further GO enrichment analysis and KEGG pathway analysis indicated that GD induced an obvious alteration of microglial phenotype in AD mice and several differentially expressed genes, some of which were involved in necroptosis process and human neurogenerative diseases. The microglial phenotype exhibited a predominant M1 phenotype and M2 polarization was inhibited in the hippocampus of GD model mice. Microglia M1 phenotype increased the expression of inflammatory factor including TNFα, iNOS, and IL-1β, which could induce neuroinflammation and neuronal damage [70]. Furthermore, TNF as a upstream regulator of necroptosis can promote RIPK/MLKL-mediated necroptosis and induce neuronal loss [26]. Though the total levels of RIPK/MLKL proteins did not change in the hippocampus of GD model mice, we found an increase in p-RIPK3 and p-MLKL levels and its interaction indicating GD-induced necroptosis activation. [25, 26]. When comparing our results to those of previous studies on necroptos is involving the RIPK1/RIPk3/MLKL pathway, our observations demonstrated that RIPK1 is not essential for activation of RIPK/MLKL-induced necroptosis in microglia of GD mice in our study [26]. In addition, we provide evidence and support that GD activates RIPK3 to induce necroptosis by a RIPK3/MLKL dependent mechanism, which was in correspondence with previous report about two distinct necroptotic pathways [71].

Even though we did not replicate the previously reported effects of THs on APP gene expression, we found that GD induced a significant increase in the levels of both soluble and insoluble Aβ_40_ and Aβ_42_ with a higher expression of BACE1 and increased β-secretase activity in AD mice in vivo. Furthermore, in vitro, T3 induced microglia necroptosis activation and inhibited microglial Aβ phagocytosis in primary microglial cells. Interestingly, inhibition of necroptosis exerts a neuroprotective effect on decreasing Aβ accumulation to some extent in vivo and in vitro.

**Fig. 7:**
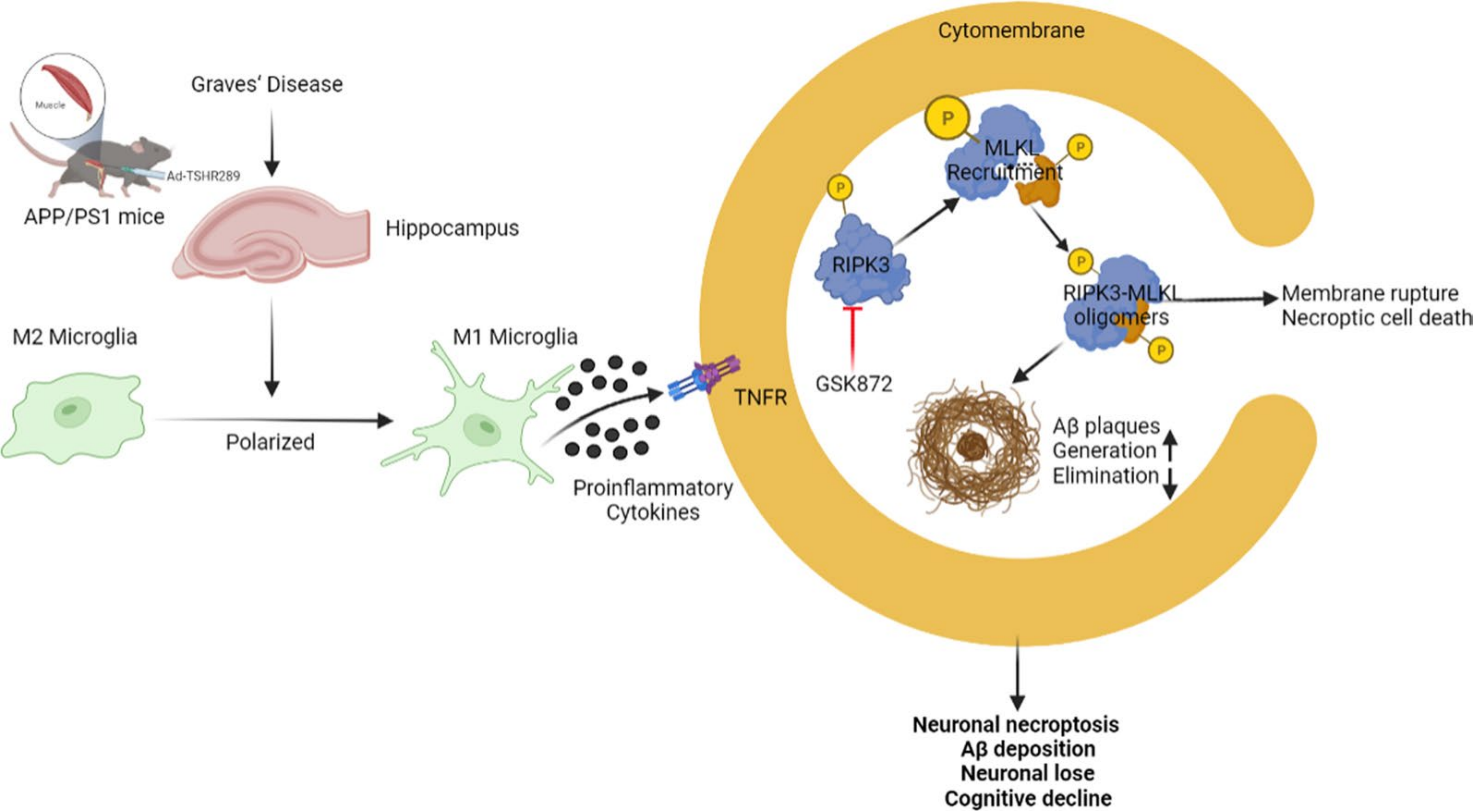
Schematic diagram of the proposed pathological mechanism underlying GD in AD model mice involving microglia-mediated necroptosis and neuroinflammation

In conclusion, our findings suggest that GD hyperthyroidism aggravates cognitive deficits and Aβ deposition in mice by inducing neuroinfammation and RIPK3/MLKL-mediated necroptosis in the brain. Moreover, inhibition of RIPK3 can inhibit Aβ accumulation and neuronal loss suggesting that these strategies can protect neurons and inhibit amyloid pathology in hyperthyroidism-induced cognitive decline (Fig. 7).

### Supplementary Information

**Additional file 1:** The image of full gel and blot.

## Data Availability

The data supporting the findings of this study are included in the supplemental material. Additional data are available from the corresponding author on request. No data are deposited in databases.

## Abbreviations

TSH: Thyrotropin
T3: Triiodothyronine
TSHR: Thyrotropin receptor
GD: Graves’ disease
AD: Alzheimer’s disease
A β: Amyloid-β
RIPK 1/3: Receptor-interacting serine/threonine protein kinase 1/3
MLKL: Mixed lineage kinase domain-like pseudokinase
CSF1R: Colony-stimulating factor 1 receptor
BACE1: Amyloid-β precursor protein-cleaving enzyme 1
iNOS: Inducible nitric oxide synthase
IL-1 β: Interleukin-1 β
IBA1: Ionized calcium binding adapter molecule 1
TNFα: Tumour necrosis factor α
UMAP: Uniform manifold approximation and projection
DEGs: Differentially expressed genes
GO: Gene ontology
KEGG: Kyoto Encyclopedia of Genes and Genomes
TT4: Total thyroxine
TRAb: TSH receptor antibody
AST: Aspartate aminotransferase
ELISA: Enzyme-linked immunosorbent assay
MWM: Morris water maze
MRI: Magnetic resonance imaging
APP/PS1: Amyloid precursor protein/prosenoxin 1
Nec-1s: Necrostatin-1s
TEM: Transmission electron microscopy
