# Supplementary material for: The effect of hyperthyroidism on cognitive function, neuroinflammation, and necroptosis in APP/PS1 mice
Source: J Transl Med. 2023 Sep 22;21:657. doi: 10.1186/s12967-023-04511-x (PMC10517505; doi:10.1186/s12967-023-04511-x)
Supplement: Supplementary file 1 — Additional file 1: The image of full gel and blot. [file 12967_2023_4511_MOESM1_ESM.pdf]

Supplementary Materials for

The effect of hyperthyroidism on cognitive function, neuroinflammation, and necroptosis in APP/PS1 mice

Kai Lou[1], Shudong Liu[2], Fengxia Zhang[3],Wenxiu Sun[4], Xinhuan Su[5],Wenkai Bi[6,7,8,9], Qingqing Yin[10], Yaxin Qiu[6,7,8,9], Zhenyuan Zhang[6,7,8,9], Mengzhe Jing[6,7,8,9], Shizhan Ma[6,7,8,9*]

[*]Corresponding author Email: mashizhan@sph.com.cn

Supplemental Experimental Procedures

Chemicals and reagents

Cell culture reagents and fetal bovine serum (FBS) were purchased from Gibco BRL (Carlsbad, CA, USA). GSK872 (a RIPK3 inhibitor) and Aβ1-42 peptide were purchased from Sigma -aldrich (St Louis, MO, USA).Rabbit anti-phospho-Ser166-RIPK1, anti-phospho-Ser232-RIPK3, anti-phospho-Ser358-MLKL, and anti-amyloid-β antibodies were purchased from Cell Signaling (Beverly, MA, USA). Rabbit anti-RIPK1, anti-RIPK3, anti-MLKL, anti-ionized calcium binding adapter molecule 1(IBA1), anti-amyloid-β precursor protein-cleaving enzyme 1 (BACE1), anti-inducible nitric oxide synthase (iNOS) and anti-Arginase1 antibodies were purchased from ABclonal Biotech (Wuhan, China). Mouse anti-GAPDH and anti-β-actin antibodies were purchased from Proteintech (Rosemont, IL, USA). Horseradish peroxidase (HRP)-conjugated anti-mouse and anti-rabbit antibodies were purchased from Jackson ImmunoResearch (West Grove, PA, USA).

Cell culture and microscopic examination and library construction

Frozen hippocampal cells were rapidly thawed and examined by microscopy after 0.4% Trypan blue staining. When the viability of the cells was higher than 80%, library construction was performed.

A single-cell library was constructed using the Chromium^{TM} Controller and Chromium^{TM} Single Cell 3' Reagent Version 2 Kit (10 x Genomics, Pleasanton, CA). Briefly, single cells, reagents and gel beads containing barcoded oligonucleotides were encapsulated into nanolitre gel beads in emulsion (GEMs) using GemCode Technology. Lysis and barcoded reverse transcription

of polyadenylated mRNA from single cells was performed within each GEM. After reverse transcription, the GEMs were washed, and the cDNA was amplified. The cDNA was fragmented, the fragment ends were repaired, and A-tails were added to the 3' end. The adaptors were ligated to fragments selected by double-sided solid-phase reversible immobilization (SPRI). Another double-sided SPRI selection was carried out after sample index PCR. The quality of the final library was assessed by checking the distribution of the fragment size using an Agilent 2100 bioanalyzer, and the library was quantified using real-time quantitative PCR (QPCR) (TaqMan Probe). The final products were sequenced using the Illumina HiSeq 4000 or Xten platform (BGI-Shenzhen, China).

Morris water maze (MWM)

The MWT test was performed as described previously. [1] The mice were habituated to the apparatus on day 0. Training trials were employed over 4 consecutive days (day 1–day 4), and the mice underwent the test three times a day. The location at which the mice were placed in the pool was changed in each trial, whereas the location of the hidden platform remained unchanged. On day 5 (probe trial), 24 hours after the previous training trial, the hidden platform was removed before conducting the tests. The cut-off time was set to 60 s. The percentage of time spent in the target quadrant and the time spent in the area where the hidden platform had been located were recorded to evaluate spatial memory capacity.

Novel object recognition test

The novel object recognition test is a behavioral test that is commonly used to investigate

various aspects of mouse learning and cognition while being less stressful to the animals.[2] This test was performed over 3 days: the habituation day, training day, and testing day. On the habituation day, each mouse was placed in the middle of an open arena and allowed to freely explore for 10 min. On the training day, two identical objects were placed in opposite quadrants of the apparatus, and the mice were allowed to freely explore the objects for 10 min. On the testing day, one familiar object used on the training day and one novel object were placed in the same arena, and the mice were allowed to explore the objects. When both objects had been explored for 20 s or when 10 min had elapsed, the test was stopped. The discrimination index, which was calculated as the interaction time with the novel object divided by the interaction time with the novel object and the familiar object x 100, was calculated for each mouse.

Y maze test

The Y-maze test was to assess the short term spatial memory ability of the mice.[3, 4] Each mouse was placed in one of the three arms and allowed to explore freely the maze for 10 min. An alternation was defined as successive entry into the three different arms on overlapping triplets. Alternation behaviour (%) was calculated as the ratio of actual alternations to possible alternations (defined as the number of arm entries minus two).

Congo red staining

Congo red staining was used to determine whether tissue slices contained amyloid.[5] Brain sections (20 mm) were stained with Congo red working solution (NaCl-saturated 80% ethanol

with 0.01% NaOH) for 10 min, incubated with solution B (Sigma–Aldrich, USA) for 35-40 min, dehydrated in 95% alcohol, incubated three times in xylene for 15 min, cleared in xylene and mounted with resinous mounting medium. Under a light microscope, amyloid deposits appeared red to orange.

Nissl staining

Nissl staining was used to visualize Nissl bodies in neurons. Staining was performed according to a previously described method.[4] The brain sections were rehydrated with distilled water before Nissl staining. After staining in 0.1% cresyl violet working solution (Sigma–Aldrich, USA) for 10 min, the sections were washed and then dehydrated with 95% ethyl alcohol for 8 s. The slices were dehydrated in 70% ethyl alcohol and 100% ethyl alcohol twice for 5 min each and cleared using xylene. Photographs of the brain sections were taken with a slide-scanning system (Tissue FAXS PLUS, Vienna, Austria).

Analysis of thyroid and liver function

The serum levels of total thyroxine (TT4) and thyrotropin (TSH) in the mice were quantitatively measured using enzyme-linked immunosorbent assay (ELISA) kit (Cusabio, Wuhan, China). The procedure of ELISA was conducted as described previously. [6] The sensitivities of the TT4 and TSH assays were 20 ng/ml and 0.15 μIU/ml, respectively, and the plate absorbance value was detected at 450 nm wavelength.

TSH receptor antibody (TRAb) and aspartate aminotransferase (AST) in the mice were quantitatively measured using ELISA kit (Jianglai Biotech, Shanghai, China) and a SpectraMax

microplate reader (Molecular Devices, Sunnyvale, CA, USA) according to the manufacturer's instructions. The sensitivities of the TRAb and AST assays were 0.2IU/L and 0.3 IU/L, respectively.

Magnetic resonance imaging (MRI)

Mice (12 months old) were imaged using an MRI scanner with a magnetic intensity of 1.0 T (Aspect-M3) and a gradient echo sequence (TR/TE=3000/75.0, ST/spacing=2.00/2.10 mm, FOV=30×30 mm, matrix=256×128, NEX=8, ETL=12, resolution=156 μm, imaging time =10 min).

Tissue sample preparation

After the behavioural experiments (the 36th week) were completed, brain tissue samples were collected. The mice were starved for 8 hours and then anaesthetized with isoflurane. Once anaesthetized, the mice were perfused with 0.9% NaCl. The hippocampus and cortex were separated to obtain samples for Western blotting. The samples were quickly stored in liquid nitrogen in a −80°C freezer before use. For immunofluorescence, the brains tissues were fixed by 4% paraformaldehyde in 4°C overnight followed by paraffin-embedding and serially sectioned at 20 μm thickness.

Western blotting

Western blotting was performed according to a previously described method.[7] Briefly, brain tissues were homogenized in ice-cold lysis buffer containing fresh protease and phosphatase

inhibitor cocktail. After incubation for 30 min on ice, the lysates were centrifuged for 30 min (4°C, 12,000 × g), and the protein content in the supernatant was measured by Bicinchoninic Acid assay. The protein samples were then subjected to sodium dodecyl sulphate- polyacrylamide gel electrophoresis (SDS–PAGE) and transferred onto polyvinyl difluoride (PVDF) membranes. The membranes were subsequently probed overnight with appropriate primary antibodies such as phospho-Ser166-RIPK1, phospho-Ser232-RIPK3, phospho-Ser358-MLKL, RIPK3, MLKL, IBA1, Aβ, BACE1 antibodies, and GAPDH antibody. The corresponding HRP-conjugated secondary antibodies were incubated for 1 hour. The protein bands were developed using enhanced chemiluminescence (ECL) (Millipore, Germany) and visualized with an Amersham Imager 680 system.

Immunoprecipitation

Immunoprecipitation is conducted as described previously.[8] Hippocampal tissues were lysed in lysis buffer containing phosphatase and protease inhibitors. The cell lysates were incubated with protein G agarose beads at 4°C for 4 hours. An MLKL antibody was added in lysis buffer for 4 hours at 4°C. The supernatants were immunoprecipitated with the antibody-coupled beads at 4°C overnight. The next day, the immunoprecipitated complexes were centrifugated and washed, andthe supernatant samples were subsequently subjected to routine western blotting analysis as described above.

Quantitative PCR (QPCR)

Quantitative PCR was performed using PrimeScript RT reagent Kit and SYBR Premix Ex Taq

II (TaKaRa, Kusatsu, Japan) in a LightCycler 480 instrument (Roche). The $2^{-\Delta\Delta t}$ method was used to analyze the targeted gene expression. All primers used for QPCR are shown in Supplementary Materials Table S1.

Thyroid histology

The formalin-fixed paraffin-embedded thyroid tissues were sectioned into 5-μm-thick sections, and fixed with tissue fixating solution for 15min. Subsequently, the sections were stained with Hematoxylin and Eosin (H&E) using standard experimental procedures. Microscope inspection, image acquisition and analysis were processed.

Immunofluorescence

Mouse brain cryosections (20 μm) were fixed in acetone, and antigen retrieval was performed in citrate buffer. The sections were incubated in blocked buffer, and probed overnight with p-RIPK1, p-RIPK3, p-MLKL, NeuN, IBA1, Aβ, iNOS and Arginase 1 primary antibodies, and then incubated with corresponding secondary antibodies for 1 hour. 4',6-Diamidino-2-Phenylindole (DAPI) was acted as a nuclear counterstain. The sections were imaged using a fluorescence microscope (Zeiss AxioImager.A2, Wetzlar, Germany).

Enzyme linked immunosorbent assay (ELISA)

The levels of $A\beta_{40}$, $A\beta_{42}$ (Thermo Fisher, Waltham, MA, USA), and β-secretase activity (Abcam, Cambridge, UK) in mouse hippocampal samples were analysed using ELISA kits according to the manufacturer's instructions. A SpectraMax microplate reader (Molecular Devices, Sunnyvale, CA,

USA) was utilized to monitor the absorbance.

Primary microglial culture

Primary microglial cultures were performed as reported previously.[9] Briefly, the hippocampal tissues were removed from newborn APP/PS1 mice. The mixed glial cells were trypsinized and homogenized. The primary mixed glial cells were cultured for 16 days and isolated using a cell isolation kit (StemCell Technologies,Vancouver, British Columbia, Canada)according to the manufacturer's instructions. The oligomeric $A\beta_{1-42}$ peptide was prepared as described previously.[10] The primary microglia was treated with oligomeric $A\beta_{1-42}$ for 3 hours at a concentration of 5μmol/L.

Supplementary Figure legends

Fig S1 (A) Representative thyroid histology from APP/PS1 and APP/PS1+GD mice (×40 magnification). (B) MR images of brain tissue from GD model mice and control mice.

Fig S2 (A) Quantification of Aβ protein load in (2B). (B) Quantification of the NeuN-positive cell density in the cortical region in mice in (2C). (C) Representative images of NeuN immunostaining and quantification in the CA1 and CA3 subfields and DG of the hippocampus in the indicated mice. Scale bar, 200 μm. (D) The number of neurons in the CA1 and CA3 subfield and DG of the hippocampus and the cortex in (2D).Unpaired Student's *t*-test, ⋆p<0.05 compared to control mice. The values are presented as the mean ± SEM (n = 3/group). N.S.: no significance.

Fig S3 (A) Quantification of IBA1 protein in (3D). (B) Quantification of the IBA1-positive cell in (3E). (C) Heatmap showing the top 10 DEGs in hippocampus 19 clusters.

Fig S4 The specific inhibitor of RIPK1, Nec-1s, or vehicle was delivered to APP/PS1 mice and GD model mice. (A) Immunostaining of p-RIPK3 in the hippocampus of the indicated mice. Scale bar, 200 μm. (B) (Top) Coimmunostaining using an anti-p-RIPK1 antibody, anti-p-RIPK3 antibody and anti-IBA1 (a microglial marker) antibody in the hippocampal region in GD model mice. Scale bar, 200 μm. (Bottom) Higher magnification images. Scale bar, 20 μm. ⋆p<0.05 compared to control mice. The values are presented as the mean ± SEM (n = 3/group). N.S.: no significance.(C) Densitometric quantification of p-RIPK3, p-MLKL, and Aβ protein levels in Fig 5A. (D) The serum levels of TT_4 (D), TSH (E), TRAb (F), and AST (G) in GSK872-treated

APP/PS1 mice with GD. N.S.: no significance. (H) Densitometric quantification of p-RIPK3 and p-MLKL protein levels in Fig 5D. (I) Quantification of p-RIPK3 levels by immunofluorescence staining (Fig 5E). (J) Quantification of p-MLKL levels in Fig 5F. (K) Densitometric quantification of BACE1 and Aβ protein levels in (Fig 6F). (L) Densitometric quantification of p-RIPK3 and p-MLKL protein levels in (Fig 6G). (M, N) The area of NeuN-positive cells (M) and p-MLKL-positive cells (N) in (6I), One-way ANOVA followed by Tukey test. * $p<0.05$ compared to control mice. The values are presented as the mean ± SEM (n = 6/group). N.S.: no significance.

Fig S5 Behavioral performance in Morris Water Maze (MWM) of GD model mice and GSK872-treated GD model mice. (A) Latency to target in the MWM test (n = 6/group). (B) Representative tracings of GD model mice and GSK872-treated GD mice in the probe trial. (C) The time spent in the target quadrant and the number of crossing the platform (D) in the probe trial.

Table S1 The primers used for quantitative RT–PCR.

	Sequence (5' -> 3')	
	Forward Primer	Reverse Primer
Tnfα	CAGGCGGTGCCTATGTCTC	CGATCACCCCGAAGTTCAGTAG
Inos	ACATCGACCCGTCCACAGTAT	CAGAGGGGTAGGCTTGTCTC
Il-1β	TTCAGGCAGGCAGTATCACTC	GAAGGTCCACGGGAAAGACAC
Arginase1	TGTCCCTAATGACAGCTCCTT	GCATCCACCCAAATGACACAT
Il-4	CCCCAGCTAGTTGTCATCCTG	CCCCAGCTAGTTGTCATCCTG
Cd206	GCTTCCGTCACCCTGTATGC	TCATCCGTGGTTCCATAGACC

Fig S1

A

APP/PS1 APP/PS1+GD

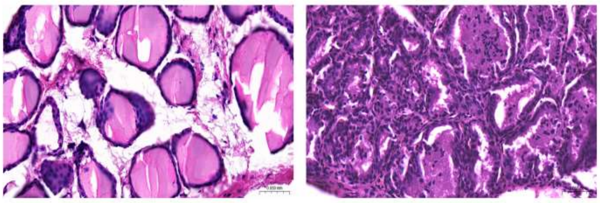

B

APP/PS1 APP/PS1+GD

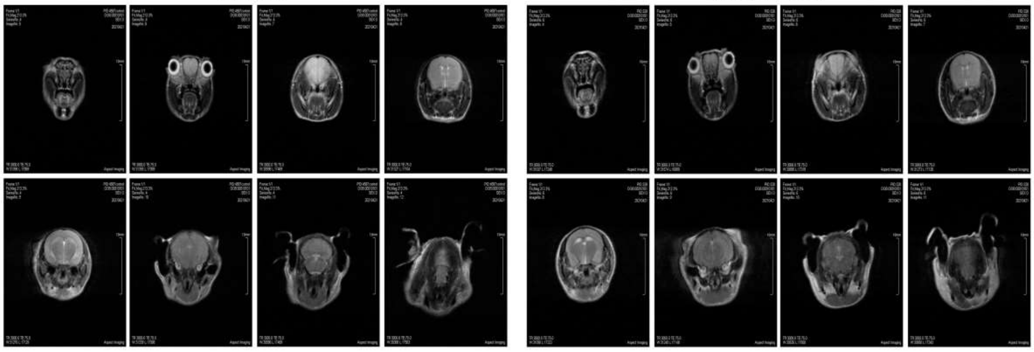

Fig S2

A

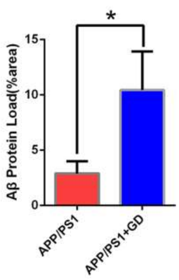
*
Aβ Protein Load(%area)
15
10
5
0
APP/PS1
APP/PS1+GD

B

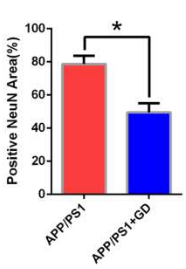
*
Positive NeuN Area(%)
100
80
60
40
20
0
APP/PS1
APP/PS1+GD

C

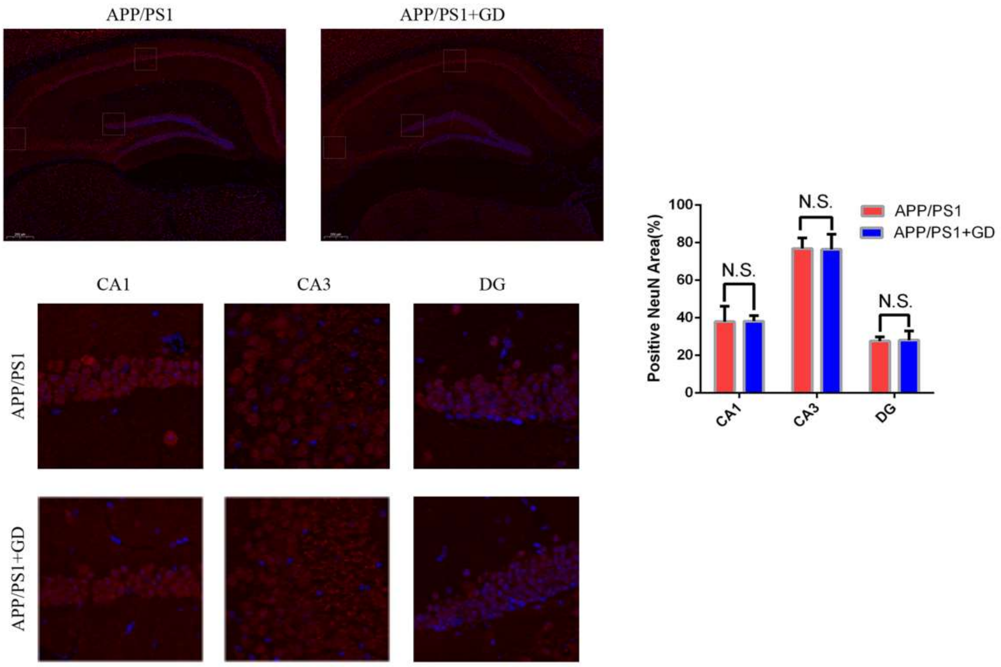
APP/PS1
APP/PS1+GD
CA1
CA3
DG
APP/PS1
APP/PS1+GD
Positive NeuN Area(%)
100
80
60
40
20
0
N.S.
N.S.
N.S.
APP/PS1
APP/PS1+GD
CA1
CA3
DG

D

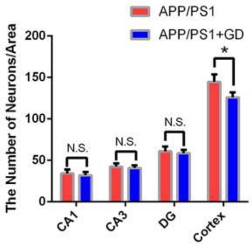
APP/PS1
APP/PS1+GD
The Number of Neurons/Area
200
150
100
50
0
N.S.
N.S.
N.S.
*
CA1
CA3
DG
Cortex

Fig S3

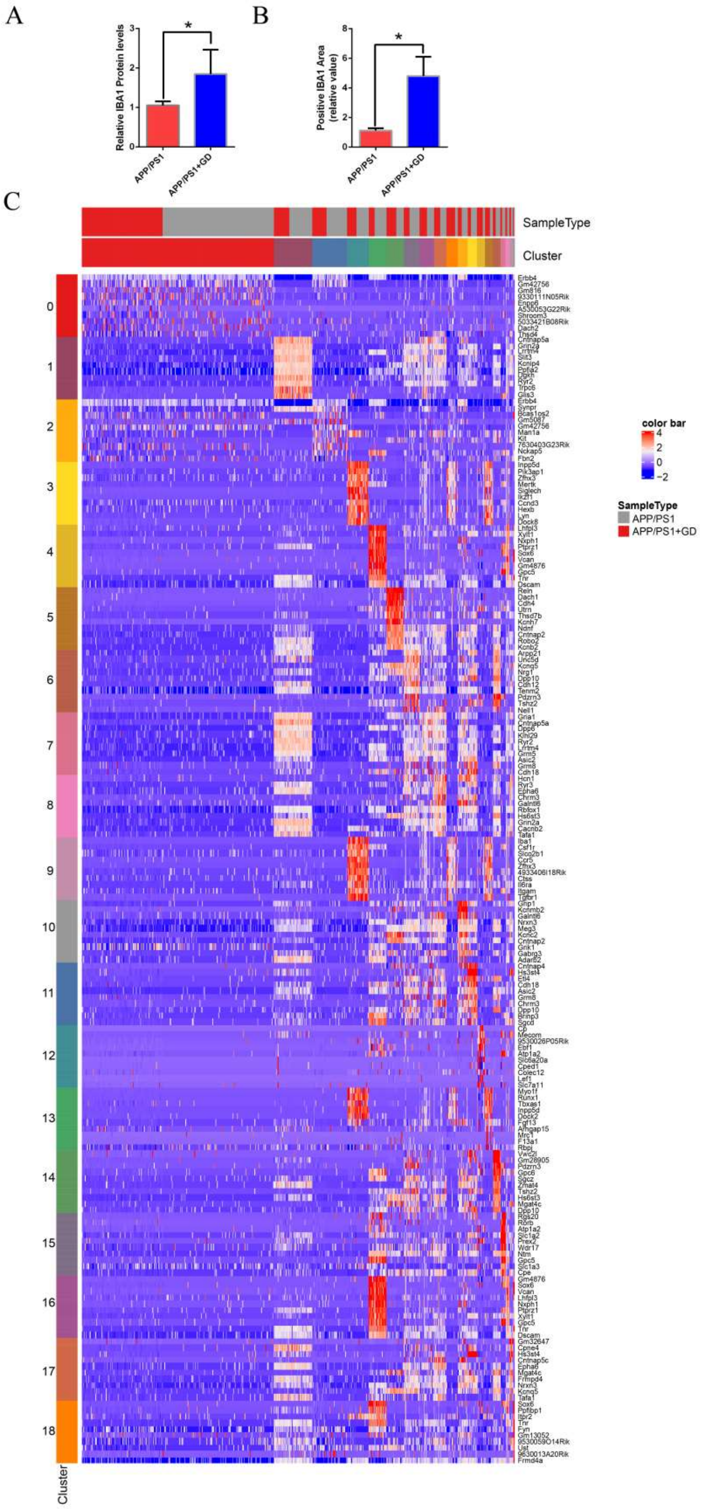
A
Relative IBA1 Protein levels
*
APP/PS1
APP/PS1+GD
B
Positive IBA1 Area (relative value)
*
APP/PS1
APP/PS1+GD
C
SampleType
Cluster
color bar
SampleType
APP/PS1
APP/PS1+GD
Cluster

Fig S4

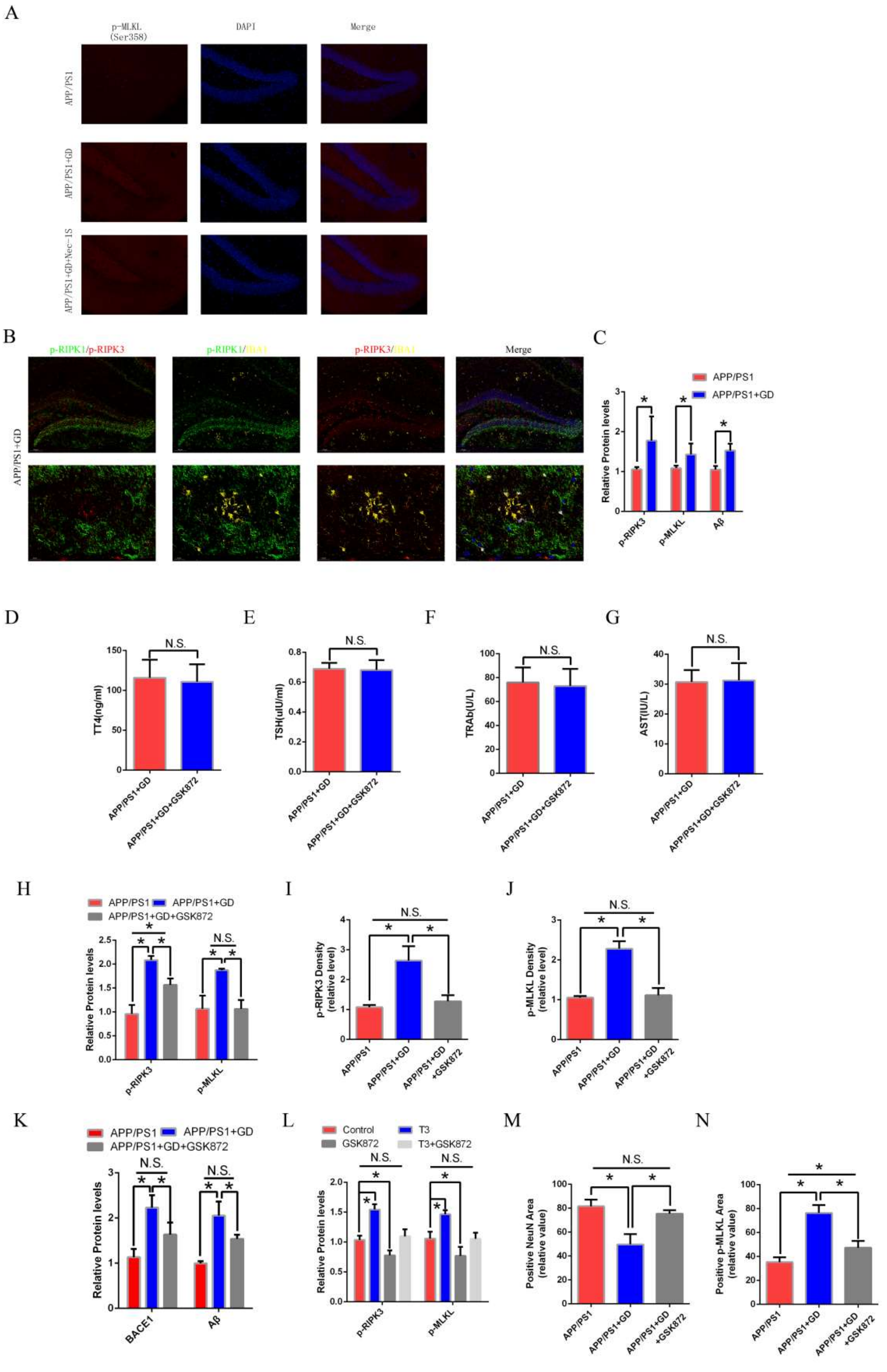

A
p-MLKL (Ser358)
DAPI
Merge
APP/PS1
APP/PS1+GD
APP/PS1+GD+Nec-1S
B
p-RIPK1/p-RIPK3
p-RIPK1/IBA1
p-RIPK3/IBA1
Merge
APP/PS1+GD
C
APP/PS1
APP/PS1+GD
Relative Protein levels
p-RIPK3
p-MLKL
Aβ
D
TT4(ng/ml)
E
TSH(uIU/ml)
F
TRAb(U/L)
G
AST(IU/L)
N.S.
APP/PS1+GD
APP/PS1+GD+GSK872
H
APP/PS1
APP/PS1+GD
APP/PS1+GD+GSK872
Relative Protein levels
I
p-RIPK3 Density (relative level)
J
p-MLKL Density (relative level)
K
BACE1
L
Control
T3
GSK872
T3+GSK872
M
Positive NeuN Area (relative value)
N
Positive p-MLKL Area (relative value)

Fig S5

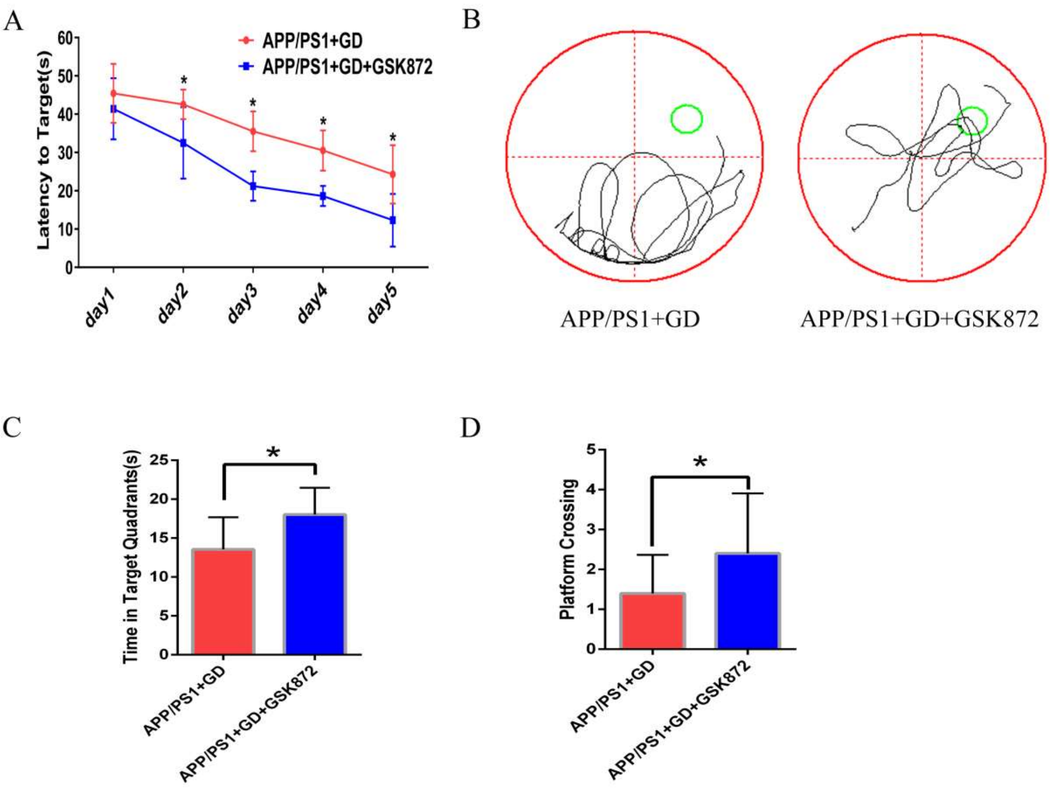
A
B
C
D
Latency to Target(s)
60
50
40
30
20
10
0
day1
day2
day3
day4
day5
APP/PS1+GD
APP/PS1+GD+GSK872
*
APP/PS1+GD
APP/PS1+GD+GSK872
Time in Target Quadrants(s)
25
20
15
10
5
0
APP/PS1+GD
APP/PS1+GD+GSK872
Platform Crossing
5
4
3
2
1
0
APP/PS1+GD
APP/PS1+GD+GSK872

References

1. Murayama MA, Arimitsu N, Shimizu J, Fujiwara N, Takai K, Okada Y, et al. Dementia model mice exhibited improvements of neuropsychiatric symptoms as well as cognitive dysfunction with neural cell transplantation. Exp Anim. 2021;70(3):387-97. doi: 10.1538/expanim.21-0008.

2. Bahn G, Park J-S, Yun UJ, Lee YJ, Choi Y, Park JS, et al. NRF2/ARE pathway negatively regulates BACE1 expression and ameliorates cognitive deficits in mouse Alzheimer's models. Proceedings of the National Academy of Sciences. 2019;116(25):12516-23. doi: 10.1073/pnas.1819541116.

3. Lalonde R. The neurobiological basis of spontaneous alternation. Neuroscience & Biobehavioral Reviews. 2002;26(1):91-104. doi: 10.1016/s0149-7634(01)00041-0.

4. Luan S, Bi W, Shi S, Peng L, Li Z, Jiang J, et al. Thyrotropin receptor signaling deficiency impairs spatial learning and memory in mice. Journal of Endocrinology. 2020;246(1):41-55. doi: 10.1530/joe-20-0026.

5. Wilcock DM, Gordon MN, Morgan D. Quantification of cerebral amyloid angiopathy and parenchymal amyloid plaques with Congo red histochemical stain. Nature Protocols. 2006;1(3):1591-5. doi: 10.1038/nprot.2006.277.

6. Swarnalatha NB, Roy N, Gouda MM, Moger R, Abraham A. High-fat, simple-carbohydrate diet intake induces hypothalamic-pituitary-thyroid axis dysregulation in C57BL/6J male mice. Appl Physiol Nutr Metab. 2018;43(4):371-80. doi: 10.1139/apnm-2017-0410.

7. Ofengeim D, Mazzitelli S, Ito Y, DeWitt JP, Mifflin L, Zou C, et al. RIPK1 mediates a disease-associated microglial response in Alzheimer's disease. Proceedings of the National Academy of Sciences. 2017;114(41):E8788-E97. doi: 10.1073/pnas.1714175114.

8. Li Y, Wang L, Zhou L, Song Y, Ma S, Yu C, et al. Thyroid stimulating hormone increases hepatic gluconeogenesis via CRTC2. Molecular and Cellular Endocrinology. 2017;446:70-80. doi: 10.1016/j.mce.2017.02.015.

9. Park J, Ha H-J, Chung ES, Baek SH, Cho Y, Kim HK, et al. O-GlcNAcylation ameliorates the pathological manifestations of Alzheimer's disease by inhibiting necroptosis. Science Advances. 2021;7(3). doi: 10.1126/sciadv.abd3207.

10. Kim T, Vidal GS, Djurisic M, William CM, Birnbaum ME, Garcia KC, et al. Human LilrB2 Is a β-Amyloid Receptor and Its Murine Homolog PirB Regulates Synaptic Plasticity in an Alzheimer's Model. Science. 2013;341(6152):1399-404. doi: 10.1126/science.1242077.
